# Mitotic Recombination and Adaptive Genomic Changes in Human Pathogenic Fungi

**DOI:** 10.3390/genes10110901

**Published:** 2019-11-07

**Authors:** Asiya Gusa, Sue Jinks-Robertson

**Affiliations:** Department of Molecular Genetics and Microbiology, Duke University Medical Center, Durham, NC 27710, USA; asiya.gusa@duke.edu

**Keywords:** mitotic recombination, pathogenic fungi, adaptation, *Candida*, *Cryptococcus*, asexual reproduction, gene rearrangements, genome diversity, DSB repair, microevolution

## Abstract

Genome rearrangements and ploidy alterations are important for adaptive change in the pathogenic fungal species *Candida* and *Cryptococcus*, which propagate primarily through clonal, asexual reproduction. These changes can occur during mitotic growth and lead to enhanced virulence, drug resistance, and persistence in chronic infections. Examples of microevolution during the course of infection were described in both human infections and mouse models. Recent discoveries defining the role of sexual, parasexual, and unisexual cycles in the evolution of these pathogenic fungi further expanded our understanding of the diversity found in and between species. During mitotic growth, damage to DNA in the form of double-strand breaks (DSBs) is repaired, and genome integrity is restored by the homologous recombination and non-homologous end-joining pathways. In addition to faithful repair, these pathways can introduce minor sequence alterations at the break site or lead to more extensive genetic alterations that include loss of heterozygosity, inversions, duplications, deletions, and translocations. In particular, the prevalence of repetitive sequences in fungal genomes provides opportunities for structural rearrangements to be generated by non-allelic (ectopic) recombination. In this review, we describe DSB repair mechanisms and the types of resulting genome alterations that were documented in the model yeast *Saccharomyces cerevisiae*. The relevance of similar recombination events to stress- and drug-related adaptations and in generating species diversity are discussed for the human fungal pathogens *Candida albicans* and *Cryptococcus neoformans*.

## 1. Introduction

In pathogenic fungi, alterations to the genome during mitotic cell division provide the means for rapid evolutionary change, adaptability, and drug resistance during host infections [1,2,3,4,5,6]. This genomic plasticity is particularly intriguing because cells must strike a balance between adaptive instability and maintaining chromosomal integrity to survive. Genome rearrangements can be introduced during clonal reproduction in fungi in the course of DNA damage repair. In this review, we provide an overview of the types of genomic rearrangements that can occur during the repair of double-strand breaks (DSBs), as described in the model yeast *Saccharomyces cerevisiae*. We then discuss the impact of mitotic recombination and genome rearrangements on adaptive changes, species diversity, and virulence for the human pathogenic fungi *Candida* and *Cryptococcus*.

*Candida albicans* is a commensal organism and the most prevalent source of fungal infections in humans. Invasive candidiasis, primarily caused by *C. albicans*, *C. tropicalis*, and *C. glabrata*, is the most common fungal disease in hospitals. The resulting blood and deep tissue infections affect a quarter of a million people annually with about 50,000 deaths worldwide [7]. A rising number of hospital-acquired infections by *Candida* spp. are increasingly difficult to treat, including recent outbreaks by the deadly and multidrug-resistant *C. auris* [8,9]. Infection by *Cryptococcus neoformans*, acquired from environmental exposures to spores and yeast, can cause cryptococcal meningitis, which occurs primarily in acquired immune deficiency syndrome (AIDS) patients and other individuals whose immune system is compromised, such as transplant recipients receiving immunosuppressive therapy [10,11]. Cryptococcal infections are the most lethal among those caused by fungal pathogens, accounting for 15% of all AIDS-related deaths, estimated at 180,000 deaths worldwide annually [12].

For these opportunistic fungal pathogens, drug resistance or the lack of access to effective anti-fungal drugs contribute to the persistence in mortality and morbidity in human populations [5,7,9,12,13]. Research has focused on better understanding how fungal pathogens adapt and evolve in the human host to evade immune defenses and persist in the face of drug treatment. Publication of the genome sequences of *C. albicans* [14] and *C. neoformans* [15,16] and genome-wide analysis of the diversity that can arise, particularly during infection, greatly contributed to our understanding of the adaptive mechanisms of these organisms [3,5,16,17,18]. Although reproduction of pathogenic fungi is largely clonal, sexual, parasexual, and unisexual cycles of reproduction in these fungi were described [11,19]. Increasing evidence suggests that both sexual and asexual reproduction contribute to the genetic variation observed in clinical and environmental isolates [11,18,20,21,22,23,24].

During infection, where reproduction is clonal, *Candida* and *Cryptococcus* spp. are capable of rapid and significant genetic changes that enhance survival in hostile host conditions and in the presence of antifungal drugs [2,25]. A number of adaptive genetic changes are possible during mitotic growth, ranging from small changes such as single-nucleotide polymorphisms (SNPs), as well as insertions and deletions (indels), to large-scale genomic changes including chromosomal duplications, inversions, and translocations [26,27]. Loss of heterozygosity and changes in the copy number of genes due to aneuploidy (loss or gain of chromosomes) are also prevalent and can contribute to survival under stress conditions [1]. Many of these genetic changes result from the repair of DSBs following DNA damage. Both *Candida* and *Cryptococcus* contain repetitive sequences throughout the genome that can serve as templates for recombinational repair of DSBs [28,29]. The mechanisms and machinery required for such repair in *S. cerevisiae* are highly conserved among fungi and all eukaryotes [26,27,30]. Therefore, knowledge of mitotic recombination mechanisms and how genome rearrangements occur in yeast is central to understanding the mechanisms by which *Candida* and *Cryptococcus* species adapt and evolve, particularly in the context of host infection.

## 2. Sources of Mitotic DSBs

During meiosis in diploid organisms, double-strand breaks (DSBs) are produced by the topoisomerase-related Spo11 protein [31]. Repair of these programmed DSBs via recombination between homologous chromosomes generates genetic diversity and is essential for the proper segregation of homologs at the first meiotic division. During mitosis, however, DSBs are pathological rather than physiological and mostly occur in the context of DNA replication. When the replisome encounters a discontinuity on one strand of duplex DNA, it is converted to a one-ended DSB, and the corresponding replication fork collapses. The fork can be re-established through homologous recombination (HR) when the intact sister chromatid is engaged by the broken end. Alternatively, a one-ended DSB can be converted to a two-ended break if encountered by a fork moving in the opposing direction. In the context of replication, such two-ended breaks are also typically repaired by engaging the identical sister chromatid, and the result is genetically silent.

**Figure 1:**
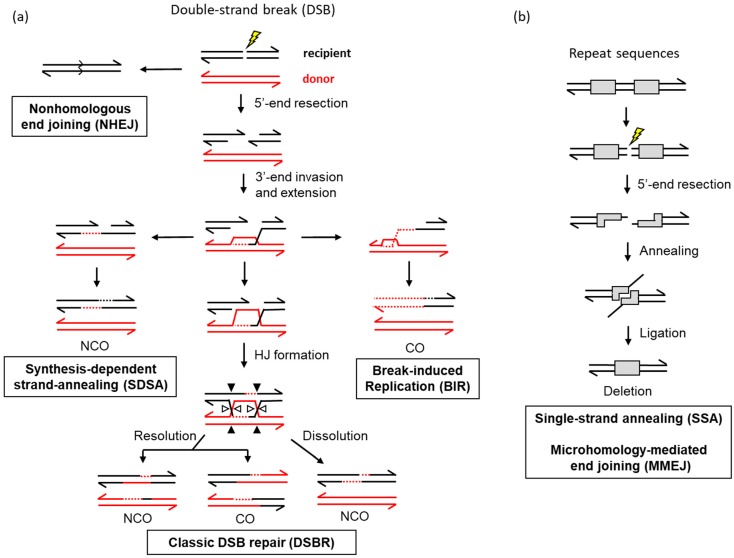
Double-strand break (DSB) repair mechanisms in *Saccharomyces cerevisiae*. Solid lines correspond to single DNA strands and dotted lines correspond to newly synthesized DNA; 3′ ends are indicated by half arrowheads. General mechanistic details are described in the text. (**a**) Unresected DNA ends are joined by non-homologous end-joining (NHEJ) while resection commits repair to a process that involves invasion of a donor repair template. During the resolution of the double Holliday junction (HJ) intermediates of the DSB repair pathway, the paired open and filled triangles reflect positions of junction cleavage and ligation. Cleavage of both junctions in either the horizontal or vertical orientation (open and closed triangles, respectively) yields non-crossover (NCO) products; cleavage in different orientations results in crossovers (COs). In break-induced replication (BIR), the invading end is extended to the end of the repair template and then is used as the template for synthesis of the complementary strand. Both new strands are on the repaired molecule, and the donor is unchanged. (**b**) Homology between direct repeats is exposed by end resection, and the annealing of the complementary strands generates tails that must be removed before ligation. Single-strand annealing (SSA) requires more homology than microhomology-mediated end-joining (MMEJ).

Although most mitotic DSBs likely occur during replication, they can also be generated by DNA damaging agents such as X-rays or reactive oxygen species. Indeed, genes whose products promote HR were initially identified in screens for radiation sensitivity in fungi (e.g., Reference [32]). Although HR is the preferred DSB pathway in the context of replication, the alternative non-homologous end-joining (NHEJ) pathway occurs efficiently when a homologous repair template is absent or when HR is disabled (reviewed in Reference [33]). NHEJ directly joins broken ends, and if the ends can be directly ligated, the repair is error-free. Presence of damaged/modified bases or sugars at the ends requires processing/removal before ligation can occur, and this frequently results in the loss or gain of DNA sequence at the break site (Figure 1). In terms of genetic consequences, HR is considered to be a high-fidelity repair process compared to NHEJ. However, it was demonstrated that DNA synthesis associated with HR is more error-prone than DNA synthesis that occurs in the context of a replication fork [34,35]. The net result is that HR is not a truly error-free process and can generate novel sequence polymorphisms, as well as rearrangements.

## 3. Mechanisms of Homologous Recombination in Yeast

The budding yeast *S. cerevisiae* was the major model organism used to define basic mechanisms of mitotic DSB (reviewed in Reference [36]). Early studies relied on spontaneous or induced recombination in diploids, with recombinants being identified as prototrophs in heteroallelic strains. The discovery that prototrophs can also be efficiently generated in haploid strains that carry an extrachromosomal plasmid or that contain heteroalleles engineered on different chromosomes greatly facilitated subsequent genetic studies [37,38,39]. Studies were further accelerated by the harnessing of meganucleases such as HO and I-*Sce*I to induce a single, targeted DSB in the yeast genome [40]. Importantly, the latter development allowed the fate of a synchronously induced DSB to be physically followed in a population of cells using Southern blots or PCR. The focus here is on what happens to DNA during repair rather than on the roles of specific genes/proteins during recombination (reviewed in Reference [36]). It should be noted, however, that the different pathways for DSB repair are often distinguished/defined by their unique genetic requirements.

DSB repair by homologous recombination requires the resection (nucleolytic degradation) of 5′ ends to (1) generate the single-stranded DNA required for a homology search, and (2) provide a 3′ end that can initiate the copying of sequence from a repair template that will span the initiating break. The broken duplex is referred to as the “recipient” allele, and the intact duplex that is copied is referred to as the “donor” allele (component single strands are black and red, respectively, in Figure 1a). A byproduct of resection is that it precludes repair via NHEJ and thereby regulates pathway choice during DSB repair (reviewed in Reference [41]). Resection is cell-cycle-regulated so that HR predominates in S and G2 phases when an identical sister chromatid is available; NHEJ is most efficient in G1. When sufficient homology is identified to support HR, the single strand invades the homologous duplex and pairs with the complementary strand to create a tract of heteroduplex DNA. The strand that has the same sequence as the invading strand is displaced as a single strand, and the resulting structure is referred to as a displacement or D-loop. The invading 3′ end is then used to prime DNA synthesis that copies the donor sequence and extends the D-loop. In the currently accepted version of the classic DSB repair (DSBR) model [42], the 3′ tail on the other side of the initiating break anneals to or “captures” the complementary, displaced strand of the D-loop [43]. This creates a second stretch of heteroduplex DNA so that the repair template is engaged by both ends of the initiating break. DNA synthesis primed from the captured end regenerates the remaining sequence removed by resection, and a Holliday junction (HJ) forms at each position where the interacting duplexes switch single strands. The HJs can be “dissolved” by migration toward each other [44] or enzymatically “resolved” by strand cleavage and ligation reactions [45]. While dissolution does not alter the genetic linkages of flanking DNA sequences/markers, resolution often does. The genetic outcome of HJ resolution can be either non-crossover or crossover product (NCO and CO, respectively). It should be noted that, if the donor and recipient duplexes contain sequence polymorphisms, these will create mismatches when complementary strands pair. Repair of such mismatches by the mismatch repair machinery can result in the acquisition of both donor strands by the recipient, which is an outcome referred to as gene conversion.

Whereas both ends of the broken DNA engage the donor duplex in the classic DSBR pathway, there are two alternative pathways in which only one end engages the donor (Figure 1a). In the synthesis-dependent strand-annealing (SDSA) [46] pathway, the expanded D-loop is dismantled by a helicase, and the extended 3′ end anneals to the complementary 3′ end on the other side of the initiating break. As in the dissolution of HJs, SDSA generates only NCO products. In the break-induced replication (BIR) pathway, extension of the invading 3′ end continues to the end of the donor chromosome to create a single CO product instead of the reciprocal products produced by classic DSBR (reviewed in Reference [47]).

In addition to recombination events that involve the invasion of a donor repair template, repeated sequences can support an additional pathway known as single-strand annealing (SSA) [48]. This typically involves direct repeats (Figure 1b), with the resection of the initiating DSB uncovering regions of complementarity that can anneal to each other. A distinguishing feature of SSA is the presence of 3′ single-stranded tails flanking the annealed region, which must be removed before ligation can restore DNA integrity. Interestingly, 3′ tail removal is catalyzed by a key endonucleolytic component (Rad1–Rad10) of the nucleotide excision repair pathway (reviewed in Reference [49]). A final pathway of DNA repair, also supported by repeat sequences, is microhomology-mediated end-joining (MMEJ) (Figure 1b). This pathway was initially defined as an alternative to classic NHEJ. MMEJ is similar to SSA and distinct from NHEJ, however, in that end resection is necessary (reviewed in Reference [50]). Unlike NHEJ, MMEJ also requires junctional homology that is in the range of 6–20 bp. The major distinction between SSA and MMEJ is that only SSA requires a protein (Rad52) to anneal the complementary segments, which are typically >30 bp.

## 4. Mitotic Recombination and Genome Alterations in *S. cerevisiae*

**Figure 2:**
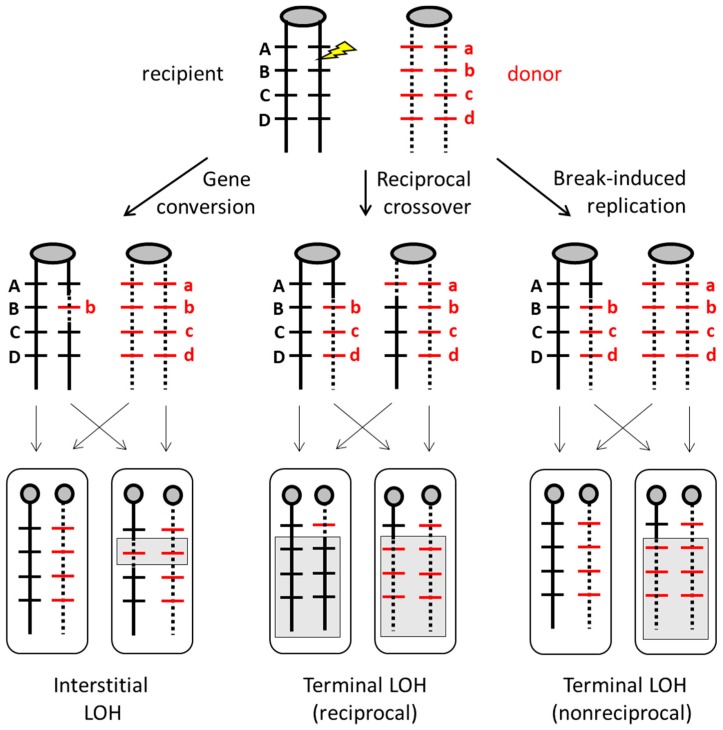
Allelic recombination and loss of heterozygosity (LOH). Replicated sister chromatids are attached at their centromeres (ovals/circles), and lines represent double-stranded DNA. The lightning bolt indicates the position of the initiating DSB, which defines the recipient molecule during repair. Black and red letters indicate heterozygous donor and recipient alleles, respectively. Thin vertical and diagonal arrows indicate segregation of sister chromatids into daughter cells, and regions of LOH are highlighted in gray boxes.

As previously noted, DSBs that occur in the context of DNA replication usually engage the identical sister chromatid and are of no genetic consequence. In diploid cells, however, a DSB can also engage the homologous chromosome by interacting with the allelic sequence that resides at the same genetic location. This can result in three types of loss of heterozygosity (LOH) when there are allelic differences between the homologs. The importance of LOH is that it can alter phenotype through the loss of dominant alleles that mask the effects of recessive alleles. In Figure 2, one homolog contains dominant alleles *A*, *B*, *C*, and *D* while the other carries recessive alleles *a*, *b*, *c*, and *d*. A DSB near the *B* allele can be repaired as a gene conversion event (classic DSBR resolved as an NCO rather than CO) that replaces it with the alterative *b* allele and results in a patch of interstitial LOH (left panel of Figure 2). Alternatively, DSB repair can lead to a reciprocal CO event between the homologs. Depending on how sister chromatids segregate at the next division, one cell may contain only dominant alleles distal to the break, while the other cell contains only recessive alleles. This is referred to as terminal LOH since it extends to the end of the chromosome (middle panel of Figure 2). Reciprocal LOH was first proposed as an explanation for so-called “twin spots” of adjacent, recessive tissue in *Drosophila* [51] and was well studied in yeast on a single chromosome arm [52,53] or genome-wide [54]. Finally, the engagement of the intact chromosome by only one end of the DSB can lead to BIR and a copying event that extends to the end the repair template. The end of the broken chromosome is lost and replaced by the end of the intact chromosome, with no genetic alteration of the repair template that is copied. The net result is the equivalent of a half-CO event and LOH in only one of the daughter cells (right panel in Figure 2).

**Figure 3:**
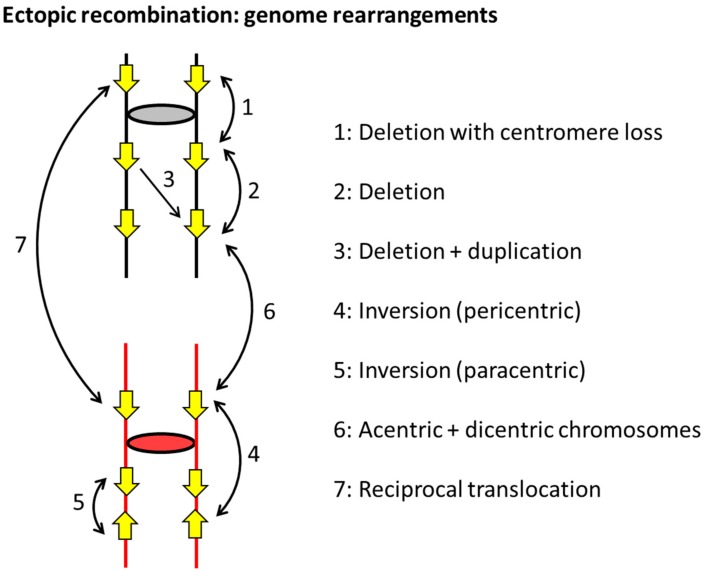
Rearrangements generated by ectopic interactions within or between chromosomes. Replicated sister chromatids of non-homologous chromosomes (one red and the other black) are attached at their centromeres (ovals/circles), and each line represents double-stranded DNA. Filled yellow arrows correspond to repeated sequences, and numbered lines with double arrowheads indicate the various types of ectopic interactions that can occur. The outcome of each type of CO-resolved interaction is indicated.

Although most mitotic recombination engages the sister chromatid (or the homolog in diploid cells), the genomes of higher eukaryotes are replete with repetitive DNA sequences that afford the opportunity for non-allele or ectopic interactions. The efficiency of HR is directly related to repeat size in *S. cerevisiae*, and as little as 100 bp is sufficient to support non-allelic or “ectopic” recombination [55]. Indeed, some of the earliest reports of spontaneous ectopic recombination involved endogenous transfer RNA (tRNA) genes that are ~150 bp [56]. Although repeats are rare in the streamlined yeast genome, the endogenous Ty1 retrotransposon, or its associated long-terminal repeats, provide portable regions of homology. Most studies of ectopic recombination, however, employed artificial repeats that allow phenotypic selection for recombination events (e.g., Reference [39]). As illustrated in Figure 3, repeats can reside on the same chromosome or on different chromosomes. When on the same chromosome, they can be in the same orientation or inverted with respect to each other (direct and inverted repeats, respectively). Although gene conversion between such repeats does not alter genome structure, it can create hybrid genes with altered or potentially new functions. 

**Figure 4:**
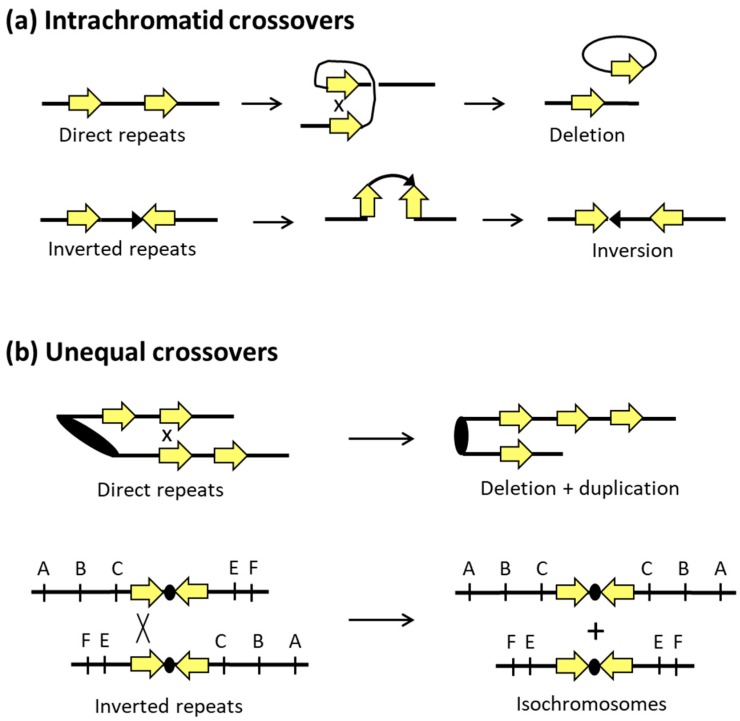
Crossover outcomes between direct and inverted repeats. (**a**) A CO between direct repeats deletes the region between the repeats and leaves one repeat on the chromosome. A CO between inverted repeats flips the orientation of the region between the repeats. (**b**) Unequal COs between direct repeats on the same arm of sister chromatids (or homologs) alter the number of repeats. If the repeats are separated by unique sequence, the intervening region is deleted in one product and duplicated in the other. An unequal CO between inverted repeats on different chromosome arms results in isochromosomes.

In contrast to gene conversion events, COs generate multiple types of genome rearrangements that are summarized in Figure 3. The outcome of an interaction reflects the relative locations of repeats, their orientations with respect to each other if they are on the same chromosome, and their relative orientations with respect to the linked centromeres. A CO between direct repeats on the same chromosome/chromatid deletes one copy of the repeat and can reflect either SSA or excision of one repeat and the intervening region as a circular piece of DNA (Figure 4a). The excised circle will be lost if it lacks an active origin of replication, but it can amplify to a high copy number if it contains an origin. Direct repeats can also be tandem, with the *CUP1* [57,58] and ribosomal DNA (rDNA) loci [59] providing examples in *S. cerevisiae* that can rapidly expand and contract to alter copy number in response to selective pressures. Recombination with tandem repeats can also give rise to extrachromosomal circles, and rDNA circles were associated with aging in yeast [60]. 

**Figure 5:**
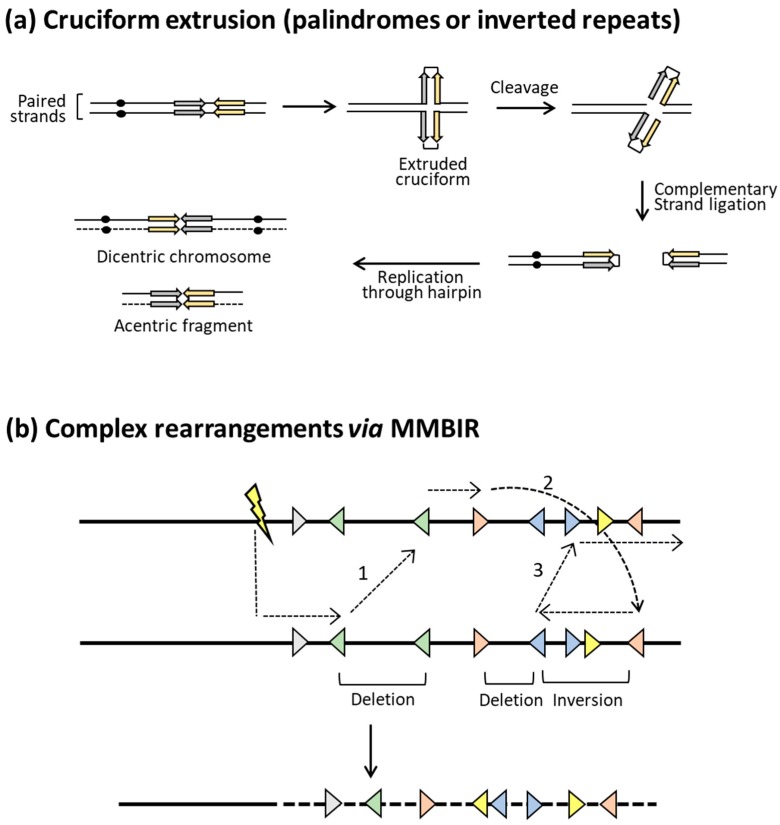
Generation of complex rearrangements. (**a**) Each line corresponds to a single DNA strand, and inverted repeats are indicated by gray and yellow arrows. Intra-strand pairing between closely/directly apposed inverted repeats generates a cruciform, the base of which resembles a Holliday junction. Cleavage creates hairpin-capped fragments, and replication through the hairpins results in large acentric and dicentric inverted chromosome fragments. The acentric fragment is unstable, while the dicentric fragment can undergo multiple rounds of bridge–breakage–fusion during subsequent cell divisions. (**b**) Lines correspond to duplex DNA, and colored triangles indicate regions of microhomology, with the relative orientation of each indicated. The lightning bolt reflects the position of the initiating DSB, and the dotted arrows indicate DNA synthesis. The numbers indicate switches to a different repair template via pairing with a region of microhomology. The final product contains multiple rearrangements.

A CO event between inverted repeats maintains the repeats, but the intervening DNA segment is inverted (Figure 4b). Recombination between inverted repeats on the same chromosome arm give rise to a paracentric inversion; recombination between repeats on different arms generates a pericentric inversion that involves the centromere (Figure 3). Depending on the size of an inversion and whether it includes the centromere, subsequent meiotic recombination between homologs may be suppressed and/or give rise to secondary types of rearrangements or instability. Inverted repeats that are very close together or directly about each other (palindromes) within a single chromosome can undergo intra-strand pairing and form an extruded, four-arm cruciform structure that resembles a Holliday junction. As illustrated in Figure 5a, cleavage at the cruciform base gives rise to hairpin-capped chromosome fragments, the replication of which produces mirror-image acentric and dicentric fragments. Acentric chromosome fragments are generally lost, while dicentric chromosomes formed in this manner initiate ongoing instability through repeated cycles of breakage–fusion–bridge [60]. Acentric and dicentric chromosomes can also be generated by CO between repeats on different chromosomes that are in different orientations relative to their centromeres (Figure 3). Finally, a CO between repeats on non-homologous chromosomes that are in the same orientation relative to their respective centromeres generates a reciprocal translocation.

Although interactions between sister chromatids are usually genetically silent, exceptions occur when repeated sequences misalign and then undergo unequal recombination (Figure 4b). As with direct repeats on homologs, a CO between misaligned sister chromatids results in a deletion of the intervening segment on one sister and its duplication on the other. When inverted repeats are on the same arm of a chromosome, an unequal CO between sister chromatids generates acentric and dicentric products. In the case of inverted repeats that flank a centromere, unequal interactions between sister chromatids can produce isochromosomes, as illustrated in Figure 4b.

In general, ectopic recombination between repeated sequences leads to relatively simple and predictable rearrangements. It should be noted that similar rearrangements can occur when there are multiple DSBs within a single genome that are joined by NHEJ (e.g., Reference [61]). Rearrangements generated by NHEJ or HR can be distinguished by the absence or presence, respectively, of homology at the junctions. It also should be noted that more complex, HR-mediated rearrangements can be a consequence of the initial homology search done by a single-stranded DNA. Recent studies in yeast demonstrated, for example, that a single tail can interact with more than one repair template in a process that is termed multi-invasion. This can lead to the joining of several nonallelic segments and chromosomal translocations [62]. In addition, there is evidence of frequent template switches during the copying of donor sequences during homologous recombination [63]. A final mechanism that can generate highly complex rearrangements involves BIR, which is inherently a non-processive process in terms of DNA synthesis. When an extending end disengages from one repair template, it can subsequently engage a different template, and this can occur multiple times. One feature of this BIR-related process is that a template switch can occur when there are only a few base pairs of homology. This particular mechanism is termed microhomology-mediated break induced replication (MMBIR) [64,65] and is illustrated in Figure 5b.

## 5. Genetic Diversity and Stress Adaptation in *Candida albicans*

*C. albicans* predominantly exists as a heterozygous diploid organism with eight chromosomes [66,67,68]. Recently, a less stable, haploid state resulting from ploidy reduction was shown to propagate within populations of diploid cells both in vitro and in vivo [69]. Aneuploidy and HR-mediated recombination events between homologous and non-homologous chromosomes are common in this organism. *C. albicans*, whose mode of reproduction is primarily clonal [70,71], is capable of mating, but does not undergo conventional meiosis. Instead, a parasexual cycle was described that involves the mating of diploid cells to form a tetraploid product, followed by recombination events and random loss of chromosomes to restore a diploid state [20,72,73,74]. Evidence indicates that the genomic diversity in *C. albicans* is largely generated during mitotic growth [28,75,76], but an additional influence of parasexual recombination on genetic diversity in this species was proposed [20,21,69,77].

The genome of *C. albicans* has a large number of repetitive sequences that contribute to the ease with which non-allelic homologous recombination events occur. Major repeat sequences (MRS) on each chromosome (partial or complete), as well as ribosomal DNA (rDNA) repeats and telomeric repeats, were implicated in genomic rearrangements that create diversity [14,75,78,79,80,81,82]. Short repetitive sequences in open reading frames (ORFs) were shown to be the primary source for generating allelic diversity in *C. albicans* by the rearrangement, addition, or deletion of repeat units within and between ORFs [83,84]. In addition, the presence of long repetitive sequences can result in inter- and intra-chromosomal recombination events. These recombination events can rapidly change the copy number and size of genes, and can result in chromosomal fusions, inversions, chimeric chromosomes, and loss of heterozygosity (LOH) [25,28,85,86]. Hundreds of previously uncharacterized long repeat sequences (~65 to 6500 bp in length, median 785 bp) were recently identified throughout the *C. albicans* genome [28]. These long repeat sequences were associated with rearrangements resulting in copy number variation, LOH, and chromosomal inversions. The majority of gene rearrangements in the clinical, environmental, and experimentally derived isolates examined had endpoints in inverted repeats that were located as far apart as 1.6 Mb on the same chromosome [28].

A major population genomics study that sequenced 182 *C. albicans* genomes from diverse origins, including all major cluster groups, described the genetic diversity among isolates as arising primarily from clonal reproduction, with some evidence of gene flow (parasexual recombination) between clusters [18]. Thousands of heterozygous SNPs and indels were identified within each *C. albicans* isolate, in addition to numerous LOH events attributed to mitotic crossovers or BIR [18]. Whole-chromosome or segmental aneuploidies were rarer, and it was suggested that the selective pressure of antifungal treatment contributes to the higher incidences of aneuploidy documented previously in clinical isolates [17]. Similarly, comparative genomic analysis of 21 clinical isolates of *C. albicans* [20] revealed that most of the genetic diversity among the isolates was consistent with changes arising from random mutations or recombination events during clonal reproduction. Variations in genotype were largely attributed to loss of heterozygosity, with many long-tract terminal LOH events consistent with either a reciprocal CO event or the nonreciprocal BIR pathway of DSB repair [20]. The only way to distinguish these pathways is through analysis of the repair template (see Figure 2). Importantly, mitotic recombination within a single genome could not explain the “mosaic” nature of some *C. albicans* isolates, whose genome showed evidence of interclade recombination resulting from a parasexual cycle [20]. 

Genomic changes that occur during infection or during short-term growth in culture are deemed microevolutionary changes. Microevolution in *C. albicans* was recently assessed using strains passaged in vitro and in vivo [3]. Both in culture and in several mouse models of infection, the majority of microevolutionary changes found were small-scale genetic changes such as SNPs and short-tract LOH (less than 10 kb in length). Interestingly, the frequency of heterozygosity gain through de novo base substitutions and indels balanced the frequency of recombination-associated LOH events, thereby maintaining the overall level of genome heterozygosity. Mutation rates (including SNPs and all genomic alterations) were higher in strains passaged through mice, and significantly higher in areas of the genome with repetitive elements such as MRS and retrotransposons. In addition, families of cell-wall adhesion genes containing internal tandem repeats were enriched for both loss- and gain-of-heterozygosity mutations. Less frequent were large-chromosomal events such as long-tract LOH and aneuploidy. In one case, trisomy of chromosome 7 was associated with a fitness advantage in the gastrointestinal tract of mice [3].

Not surprisingly, genomic changes increase when *C. albicans* is exposed to stress. LOH events, for example, are a frequent and common outcome of stress exposure in this organism, and these events increase during in vivo models of infection [3,87]. Recently, the genetic diversity arising in the mouse oral cavity during infection was examined [4]. Examples of aneuploidy and LOH were found on all chromosomes, and the occurrence of recombination events was 100 times more likely in isolates passaged through the mouse oral niche compared to isolates passaged in vitro. Previous studies identified factors such as elevated temperature (37 °C), transformation of exogenous DNA, and exposure to antifungal drugs that increase the rates of LOH [76,88].

Partial or whole-chromosome LOH in diploid organisms can result in the expression of recessive alleles (Figure 2) that have detrimental outcomes, with a classic example being loss of a tumor suppressor in the development of cancer [89]. A recent study examined the genetic outcomes of an induced DSB in *C. albicans* [90]. As in *S. cerevisiae*, repair was associated with LOH events including gene conversions, mitotic crossovers, and BIR. In addition, lethal recessive alleles were uncovered. One was an allele of the *GPI16* gene on chromosome 4 (Chr4; haplotype B), specific to the SC5314 strain [90]. The accumulation of recessive alleles may serve to limit the number of loss-of-heterozygosity events and preserve genetic diversity in *C. albicans*. However, some LOH events clearly have advantageous outcomes under certain stress conditions, such as those encountered in the host environment. One interesting example of this was the demonstrated conversion of *C. albicans* from a pathogen to a gut symbiont through the loss of Flo8 activity [91]. *FLO8* encodes a transcriptional activator essential for the yeast-to-hyphae transition, a key virulence factor in *C. albicans*. These non-filamentous homozygous *flo8* mutant strains outcompeted wild-type *C. albicans* in the gastrointestinal (GI) tract of the mouse to form a mutualistic interaction beneficial for both fungus and host [91]. As another example, partial (interstitial) LOH resulting in gene conversion of the transcription factor *EFG1* allele, was recently demonstrated while investigating the white-to-gray phenotype transition in *C. albicans* [92]. In naturally occurring isolates hemizygous for *EFG1 (EFG1^+/−^)*, loss of the functional *EFG1* allele, primarily due to gene conversion (see left panel, Figure 2), resulted in the observed white-to-gray colony transition and associated fitness advantages for *C. albicans* in the GI tract of mice [92]. Finally, fitness advantages due to LOH were also demonstrated when *C. albicans* was exposed to specific stresses in vitro. “Stress-induced LOH” can lead to the rapid adaptation of *C. albicans* in vitro via different mechanisms [76]. Whereas LOH by mitotic recombination (see Figure 2) was increased by oxidative stress, whole-chromosome LOH (due to chromosomal nondisjunction) was favored following exposure to high temperature or to the antifungal drug fluconazole (FLC) [76].

Development of drug resistance to azoles is common in fungal infections. One mechanism of FLC resistance in *C. albicans* acquired during infection involves a recombination event in which the left arm of chromosome 5 is duplicated, forming a novel isochromosomal structure [72]. The resulting duplication in copy number of the genes *ERG11* (target of FLC) and *TAC1* (transcriptional activator of efflux pumps) is associated with increased FLC resistance [86,93,94,95]. It was proposed that chromosomal breakage at the centromere of Chr5 and subsequent repair by HR at a long inverted repeat present in this region could generate this isochromosome [86] (see Figure 4b). Resistance to FLC by segmental aneuploidy of a different chromosome (Chr4) was also demonstrated in *C. albicans* [28]. In the presence of fluconazole, an FLC-sensitive strain with three copies of Chr4 evolved a novel isochromosome that resulted in FLC resistance. This isochromosome contained two right arms of Chr4, and recombination was likely facilitated by long inverted repeat sequences at the centromere. It was proposed that errors in DNA replication resulting in DSBs were more common in the centromere regions as they are the earliest sites of DNA replication initiation in *C. albicans* [28]. Alternatively, it may simply be the presence of the correct structural features to support isochromosome formation when a DSB occurs.

## 6. Genetic Diversity and Stress Adaptation in *Cryptococcus neoformans*

*Cryptococcus neoformans* is an encapsulated basidiomycete that predominantly reproduces as a haploid yeast in the environment and during infection. During its sexual cycle, *Cryptococcus* adopts a diploid, filamentous form [96]. Disease-causing *Cryptococcus* spp. were originally differentiated by antigenic variations in their polysaccharide capsules [97,98] and are now classified phylogenetically [99]. The majority (~95%) of infections that result in meningoencephalitis in immunocompromised patients are caused by *C. neoformans* (serotype A), while a smaller number of infections are caused by *C. deneoformans* (serotype D) [100]. Although these species diverged more than 18 million years ago [97], they are closely related, capable of mating and forming AD hybrids in the environment [101]. Another closely related species, *C. gattii* (serotype B) [98], was found to cause infections in immunocompetent and immunocompromised individuals [97]. 

Prior to whole-genome comparisons of cryptococcal strains, multiple studies documented karyotype variation arising in laboratory strains and during the course of infection [102,103,104,105,106,107,108]. Isolates recovered from patients with chronic AIDS infections or following passage through mice exhibited phenotypic differences in terms of virulence, growth rate, capsule size, capsule structure, and drug resistance [102,106,109]. The associated genomic rearrangements and changes in chromosome length suggested that these adaptations may be important for sustaining chronic infections and developing resistance to antifungal treatment [102,106]. Although the causes of these differences in genotype and phenotype were not determined, large-scale gene rearrangements such as translocations, deletions, and duplications could explain the observed differences in chromosome length and organization. A recent effort to compare *C. neoformans* in populations of clinical and environmental isolates from sub-Saharan Africa identified genotype and phenotype variations in virulence factors and stress-response genes associated with different lineages of the organism (VNBI and VNBII) [23]. Increased melanization and resistance to oxidative stress were found in both clinical and environmental isolates of the VNBI lineage compared to VNBII, although VNBII isolates were more prevalent among clinical isolates. A loss-of-function mutation in the transcription factor *BZP4* was linked to the absence of melanization in both lineages [23]. Recent outbreaks of *C. gattii* infections in immunocompetent populations in the Pacific Northwest of North America prompted several studies to investigate differences in genes and gene expression among the four recognized lineages (VGI, VGII, VGIII, and VGIV) in the *C. gatti* complex and between cryptococcal species [110,111,112,113]. Comparative population studies will continue to be important for linking the genetic sources of phenotype variation with differences in virulence and pathogenesis. 

Examples of enhanced virulence or resistance to drug treatment arising during cryptococcal infection continue to be found [5,114,115], and, in some cases, the cause is identified. A recent study analyzed cryptococcal isolates from the cerebrospinal fluid of patients with recurrent meningitis caused by *C. neoformans* and *C. gatti* infection [5]. A number of genetic differences were found between isolates collected during the initial diagnosis and those collected from the relapse infection. These changes included SNP variations, indels, and chromosome duplications that were either correlated or directly associated with enhanced virulence (growth at higher temperature, capsule production) and FLC drug resistance. In some cases where phenotypic differences occurred but no genetic differences were detected, epigenetic factors were inferred [5].

Although *Cryptococcus* generally exists in a haploid state, hybrid genomes and genomes with whole-chromosome duplications were documented [1]. One extreme example of whole-chromosomal duplication can occur in up to 20% of *Cryptococcus* during infection where cells increase dramatically in size and double, triple, or quadruple their chromosomal contents [116,117,118,119]. These polyploid cells, aptly named titan cells, are resistant to phagocytosis by host macrophages and are also resistant to drug treatment. Titan cells generally produce haploid or aneuploid budding progeny but do not themselves replicate as giant polyploid cells, revealing a temporary strategy to overcome environmental stress [120]. 

Variations in gene copy number through the loss or gain of chromosomes can result in differing phenotypes affecting virulence and drug resistance. For example, variations in chromosomal number in *Cryptococcus* isolated from infected AIDS patients and following passage in mice were shown to affect levels of melanin expression and other virulence traits [6,121]. Similar to FLC resistance in *C. albicans*, chromosome duplication (disomy of chromosome 1) was shown to confer FLC resistance in *C. neoformans* in vitro by increasing the copy number of *ERG11* (the target of FLC) and *AFR1* (an azole transporter) [122]. Additional duplications in other chromosomes were correlated with increased survival in the presence of increasing drug concentrations. Significantly, as concentrations of FLC were lowered and the selective pressure removed, *Cryptococcus* lost the extra chromosomes and returned to its original levels of susceptibility [122]. A recent study confirmed FLC resistance in *Cryptococcus* resulting from Chr1 disomy in human infections by genomic analysis of isolates from the cerebrospinal fluid of patients receiving FLC therapy for cryptococcal meningitis [13].

As observed in *S. cerevisiae*, variations in gene copy number can result from recombination-associated duplications. A gene amplification event unique to a *C. neoformans* subclade was recently identified as a microevolutionary adaptation conferring resistance to arsenite [123]. The gene, encoding an arsenite efflux transporter (*ARR3*), was found in tandem array (2–15 copies) in the telomeric region of chromosome 3. Exposure of *Cryptococcus* to increasing concentrations of arsenite resulted in highly resistant strains with up to 50 additional copies of the *ARR3* amplicon, analogous to *CUP1* amplification in *S. cerevisiae* [44]. Tandem arrays are thought to originate from multiple gene duplication events following DSB repair [124,125]. In *S. cerevisiae*, DNA re-replication of genes with flanking repetitive sequences was shown to induce gene amplification through non-allelic HR repair of DSBs [126].

In addition to adaptive measures in response to stress, whole-genome comparative studies uncovered the role of large chromosomal rearrangement events in the evolution and speciation of *Cryptococcus*. The cryptococcal genome has a number of repetitive sequences including rDNA repeats and repeats within the *MAT* locus. Repeat sequences are also found in transposons, which comprise 5% of the cryptococcal genome [15]. Transposons were shown to play a significant role in shaping the genome of *S. cerevisiae* [127], and their role in *C. neoformans* appears to be significant as well. The centromeres of the 14 cryptococcal chromosomes contain numerous transposon sequences [128,129] that were implicated in a number of genomic rearrangement events.

Comparative genomic analysis of the widely used laboratory strains *C. neoformans* H99 and *C. deneoformans* JEC21 identified numerous translocations, inversions, and complex rearrangements that differentiate the two species [29]. A strong association between chromosomal rearrangements and the presence of transposable elements was found, particularly near sites of proposed translocation events and complex rearrangements. Comparison of the two genomes revealed the presence of an “identity island,” a 40-kb region in the subtelomeric region of chromosome 5 of JEC21, that is nearly identical to a region in H99 on a non-homologous chromosome [130]. This region of unusual sequence identity was proposed to have been caused by non-reciprocal transfer from *C. neoformans* to *C. deneoformans* through a hybrid intermediate about two million years ago. Partial copies of the Cnl1 retrotransposon found at the boundaries of the identity islands in both genomes were the likely source of repetitive sequences that facilitated this introgression. Further analysis of the *C. neoformans* H99 strain revealed an important translocation unique to this strain [131]. Translocation between chromosomes 3 and 11 resulted in disruption of two genes affecting glucose metabolism and melanin production. Presumably, these differences result in enhanced virulence during infection. Microhomology (3 bp) at the breakpoint on each chromosome, as well as a single-nucleotide insertion preceding the region of microhomology on chromosome 11, was consistent with a non-homologous end-joining event [131], but could also reflect MMBIR. One final example of a recombination event differentiating JEC21 from H99 involves a translocation and duplication event unique to JEC21 [132]. Fusion of the telomeres of chromosomes 8 and 12, followed by breakage of the fused chromosome, resulted in segmental duplication of a 16-kb region on each chromosome. This recombination event was proposed to have occurred by meiotic recombination between subtelomeric transposon repeat sequences. However, this event may also have occurred during mitotic growth through NHEJ-mediated telomere fusion. 

NHEJ appears to be an important pathway of repair in *Cryptococcus*, despite its propensity for mutagenic outcomes. Strategies to introduce exogenous DNA for gene deletions or complementation at targeted locations showed that rates of recombination by HR in *Cryptococcus* are low and require long stretches of homologous regions (750 to 1000 bp) to maximize targeted integration [133,134]. This is in marked contrast to *S. cerevisiae*, which has an extremely high efficiency of DNA integration by HR, requiring only small regions of homology (<100 bp) [135]. Transformation of *Cryptococcus* usually results in non-targeted ectopic integration of the introduced DNA. To increase the frequency of homologous recombination for targeted gene integration, strategies to inhibit or disable NHEJ components were successfully employed [136,137].

Both NHEJ and HR-mediated pathways of DNA repair contribute to genetic variation in *Cryptococcus*. In the absence of homologous chromosomes or sister chromatids, nonallelic HR-mediated recombination is facilitated by repetitive sequences. As discussed previously, repeat sequences in transposons serve as substrates for recombination, and recombination events involving transposons contributed significantly to the evolution and genetic variation in *Cryptococcus* [111,112,113,114]. In addition, the excision of transposons from sites in the genome can cause DSBs and initiate genome instability, increasing the likelihood of mutation and gene rearrangements through DNA repair [138]. Although transposon movement is typically suppressed to prevent genome instability [139], we recently demonstrated the mobilization of transposons in *C. deneoformans* in a mouse model of infection and in response to temperature stress in vitro [140]. The movement of transposons in *Cryptococcus* in response to stress conditions may have adaptive implications for the organism not only in terms of disruption of genes or gene expression at the site of integration but also in triggering genomic rearrangements that result from repair of DSBs.

## 7. Conclusions

Mutations and recombination events resulting from DNA damage repair are a significant driver of species diversity in fungal pathogens. Although DNA damage poses a threat to genome integrity, opportunistic pathogens can exploit the resulting diversity to drive adaptation and increase survival in the host environment. Recent microevolution studies in *C. albicans* and *C. neoformans* describe both small-scale (SNPs, indels, and duplications) and large-scale genetic changes (LOH, aneuploidy) that arise during the course of infection [3,4,5]. The frequency and types of mutations and rearrangements appear to depend on the specific host niche and environmental stresses encountered. Due to the survival risks inherent in introducing genetic change, it makes sense that both *Candida* and *Cryptococcus* evolved adaptive genetic strategies that are temporary or reversible. Dynamic changes in ploidy and the loss/gain of genes in tandem repeats are examples of temporary changes that can alter the copy number and expression of genes to confer a selective advantage. Even when recombination events are irreversible (such as loss of heterozygosity), other mutations and recombination events can serve to reintroduce heterozygosity to maintain diversity in the genome [3]. The genomic plasticity of *Candida* and *Cryptococcus* enabled these species to adapt and evolve successfully in a variety of environmental and host niches.
